# Enzymes in Food Industry: Fermentation Process, Properties, Rational Design, and Applications

**DOI:** 10.3390/foods13193196

**Published:** 2024-10-08

**Authors:** Jingming Zhao, Fufeng Liu

**Affiliations:** 1 Manchester Institute of Biotechnology, School of Chemistry, The University of Manchester, 131 Princess Street, Manchester M1 7DN, UK; 2 Key Laboratory of Industrial Fermentation Microbiology, Ministry of Education, Tianjin Key Laboratory of Industrial Microbiology, College of Biotechnology, Tianjin University of Science and Technology, Tianjin 300457, China

## 1. Introduction

Enzymes play a vital role in the food industry, where they act as natural catalysts to improve production processes, enhance food quality, and increase efficiency. Recent advancements in enzyme engineering, particularly through rational design and modification, have led to significant improvements in enzyme properties such as thermostability and activity, broadening their potential industrial applications. This editorial summarizes five studies that collectively highlight the impact of enzymes in food fermentation, immobilization application, and how rational design is shaping their future use in the food industry.

## 2. Thermostable Proteases: Unlocking New Applications in Food Processing

Proteases are indispensable in the food industry, where they are used for hydrolyzing proteins in processes such as meat tenderization, dairy production, and baking. However, conventional enzymes often fail to withstand the high temperatures of industrial food processing, limiting their efficiency and cost-effectiveness. The study on serine protease PB92 from *Bacillus alcalophilus* illustrates how this challenge can be overcome. Through site-directed mutagenesis, Miao et al. [1] identified key residues that, when mutated, increased the enzyme’s thermostability by up to 31 times at 65 °C. The creation of mutants like N18L-R143L-S97A and N18L-R143L-G100A demonstrates the power of rational enzyme design, which is crucial for industrial processes that require high-temperature stability.

In food processing, this development is particularly relevant for applications that demand enzymes that can maintain activity during heat treatments, such as pasteurization or high-temperature extrusion. By ensuring that enzymes remain functional in such environments, manufacturers can streamline their processes, reduce enzyme usage, and ultimately lower production costs. Additionally, thermostable proteases offer the possibility of new product innovations, such as improved textures and flavors in protein-based foods, fermented products, and plant-based alternatives.

## 3. Xylanases in Baking: Pushing the Boundaries of Bread Quality

The use of xylanases in baking has garnered significant attention due to their ability to improve dough structure, enhance bread volume, and increase shelf life. The article on the glycoside hydrolase family 11 (GHF11) xylanase, AusM, highlights how enzyme engineering can lead to improved performance in bread-making applications. By developing variants resistant to natural inhibitors like SyXIP-I, Zhang et al. [2] ensured that the enzyme could maintain its functionality during dough preparation, contributing to better loaf volume and texture.

This advancement aligns with current trends in the food industry, where there is a growing demand for clean-label products that maintain high quality without the use of artificial additives. Xylanases enable manufacturers to reduce the need for emulsifiers and other chemical agents, promoting cleaner ingredient lists while still achieving desired product attributes. The ability to engineer xylanases that can withstand the challenges of industrial baking environments also points to future opportunities for more sustainable and cost-effective production methods, as these enzymes contribute to more efficient use of raw materials.

## 4. Applications of Engineered Enzymes in the Food Industry

In the food industry, egg white’s excellent foaming properties make it a crucial ingredient in various products like baked goods. However, even minimal contamination by egg yolk can drastically reduce the foaming ability, leading to suboptimal product quality. A recent study addresses this issue by characterizing a lipase from *Bacillus subtilis*, known as Lip-IM, which was expressed in *E. coli* and demonstrated promising results for hydrolyzing residual yolk lipids in egg whites [3]. Lip-IM showed strong hydrolytic activity, especially on long-chain fatty acids such as C16 and C18, which are abundant in egg yolk lipids. When applied to egg white contaminated with 0.5% yolk, the lipase improved foaming ability by 26% and significantly reduced the liquid precipitation rate. This makes Lip-IM a potential biocatalyst to restore the functional properties of egg white in industrial processes where contamination is inevitable. Moreover, molecular dynamics simulations revealed that Lip-IM maintained a stable conformation across various temperatures, providing insights into its structural resilience. This study paves the way for the use of lipases in improving the performance of contaminated egg whites, offering a sustainable and efficient solution for the food industry.

The immobilization of enzymes represents a critical advancement in enhancing enzyme reusability and efficiency, particularly in the food industry. A recent study explored the noncovalent immobilization of enological pectinase on magnetic-sensitive polyamide microparticles for use in wine clarification [4]. Pectinases play an essential role in breaking down pectin in grape must, thereby improving juice extraction and reducing turbidity during wine production. The study demonstrated that immobilized pectinase complexes exhibited superior catalytic performance compared to free enzymes, with more than double the specific activity towards pectin substrates. The immobilized enzymes also showed higher resistance to inhibition and could be reused for up to three cycles without significant loss of performance. This development is crucial for industrial winemaking, as it enhances enzyme recovery and reduces costs associated with enzyme replenishment. Overall, the immobilization of pectinases on polyamide microparticles opens new possibilities for more efficient and sustainable wine production. This innovative approach not only improves enzyme functionality but also contributes to the development of cleaner, greener processes in the food industry, aligning with the growing demand for sustainability.

Polysaccharides from marine algae like *Ulva lactuca* are gaining attention for their extensive health benefits, including antioxidant, antiviral, and anti-inflammatory properties. However, the low yield and high cost of extraction have limited their commercial application. A recent study explored a novel method using ultrasound-assisted multi-enzyme extraction to enhance the yield and activity of Ulva polysaccharides [5]. The study demonstrated that combining cellulase, pectinase, and protease in specific ratios significantly improved polysaccharide extraction, increasing the yield from 6.43% to over 30%. Additionally, the extracted polysaccharides exhibited strong antioxidant properties, outperforming those obtained through traditional methods. The ultrasound-assisted process enhanced cell wall degradation, which, when coupled with enzymes, enabled a more efficient and cost-effective extraction process. This approach not only improves the yield but also preserves the biological activity of the polysaccharides, making it a promising technique for industrial applications. Given the growing demand for natural bioactive compounds in food, cosmetics, and pharmaceuticals, this extraction method represents a significant advancement, offering a sustainable and scalable solution for the commercial production of Ulva polysaccharides.

## 5. Conclusions and Future Direction

The future of enzymes in the food industry is closely tied to advancements in rational design and enzyme engineering. As researchers continue to improve key properties such as thermostability and inhibitor resistance, the potential applications of these enzymes will expand, driving innovation across multiple sectors of the food industry. From fermentation processes to the production of plant-based foods and baked goods, enzymes will continue to play a pivotal role in enhancing food quality, production efficiency, and sustainability.

Moreover, the integration of computational tools into enzyme development, the application of immobilization, and ultrasound-assisted enzyme extraction technologies will further accelerate these advancements, reducing the time and costs associated with bringing new enzyme solutions to market. As the food industry increasingly focuses on sustainability and cleaner production methods, the role of engineered enzymes will become even more central to meeting these goals.

In conclusion, the studies reviewed here provide a glimpse into the future of enzyme applications in the food industry. Through rational design and the careful modification of enzyme properties, researchers are creating powerful tools that will enable more efficient, sustainable, and high-quality food production for years to come.
